# Unraveling Neurodevelopment: Synergistic Effects of Intrinsic Genetic Programs and Extrinsic Environmental Cues

**DOI:** 10.1002/advs.202414890

**Published:** 2025-05-05

**Authors:** Yanyan Wang, Shukui Zhang, Huiyang Jia, Fen Ji, Jianwei Jiao

**Affiliations:** ^1^ State Key Laboratory of Organ Regeneration and Reconstruction Institute of Zoology Chinese Academy of Sciences Beijing 100101 China; ^2^ University of Chinese Academy of Sciences Beijing 100049 China; ^3^ Beijing Institute for Stem Cell and Regenerative Medicine Beijing 100101 China

**Keywords:** angiogenesis, neurogenesis, neural stem cell, neuron, microglia

## Abstract

The development of the brain involves the proliferation of neural stem cells (NSCs) and the differentiation of neurogenic cells, including neurons, astrocytes, and oligodendrocytes. The growth and development of other cells in the brain, including microglia and blood vessels, are also crucial for maintaining normal brain function. In the first part, the intrinsic regulatory mechanisms of NSCs during neurogenesis are primarily delved into. This focused on the effects of epigenetic modifications on the proliferation and differentiation of NSCs. Additionally, the phenomenon where their own proliferative behavior leads to the activation of immune‐related genes are discussed. Furthermore, the impact of maternal immune activation on neurogenesis are explored. Finally, the reasons underlying differences in brain development between humans and mice are examined. In the second part, the development and origin of microglia, their heterogeneity during the developmental process, and the impact of microglia on the development of surrounding cells are delved into. In the third part, the relationship between the cerebrovascular system and brain development are explored. This includes the communication and interaction between blood vessels and NSCs, as well as the effects of cytokines secreted by blood vessels on synapses and the genesis of glial cells.

## Graphical abstract

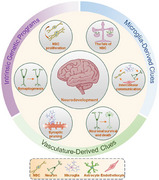

## 1 Introduction

In recent years, significant progress has been made in the field of neural development, uncovering the complex and intricate regulatory mechanisms governing the process from early neural development to system maturation. However, understanding the intrinsic and extrinsic regulatory mechanisms of neural development remains challenging due to the multi‐level, spatiotemporal dynamics involved. For example, how epigenetic modifications precisely regulate NSC fate decisions, how the specific mechanisms by which microglia influence neurogenesis, and how the neurovascular unit synergistically supports neurogenesis are still not fully understood. Meanwhile, the continuous advancement of technologies such as single‐cell sequencing and gene‐editing tools provides novel perspectives and powerful methodologies, driving the field toward new heights of discovery.

This review focuses on the intrinsic and extrinsic factors regulating neural development, aiming to integrate existing research findings with the latest advances to address current knowledge gaps, particularly in the cross‐disciplinary regulatory mechanisms of neurogenesis. For instance, the contributions of epigenetic modifications to the spatiotemporal regulation of neural development, the role of microglia in the NSC niche, and the interplay between vasculature and the nervous system during development are critical scientific questions that require further investigation. By summarizing key advancements in these areas, this review seeks to clarify future research directions and provide theoretical insights into neural development.

**Figure 1:**
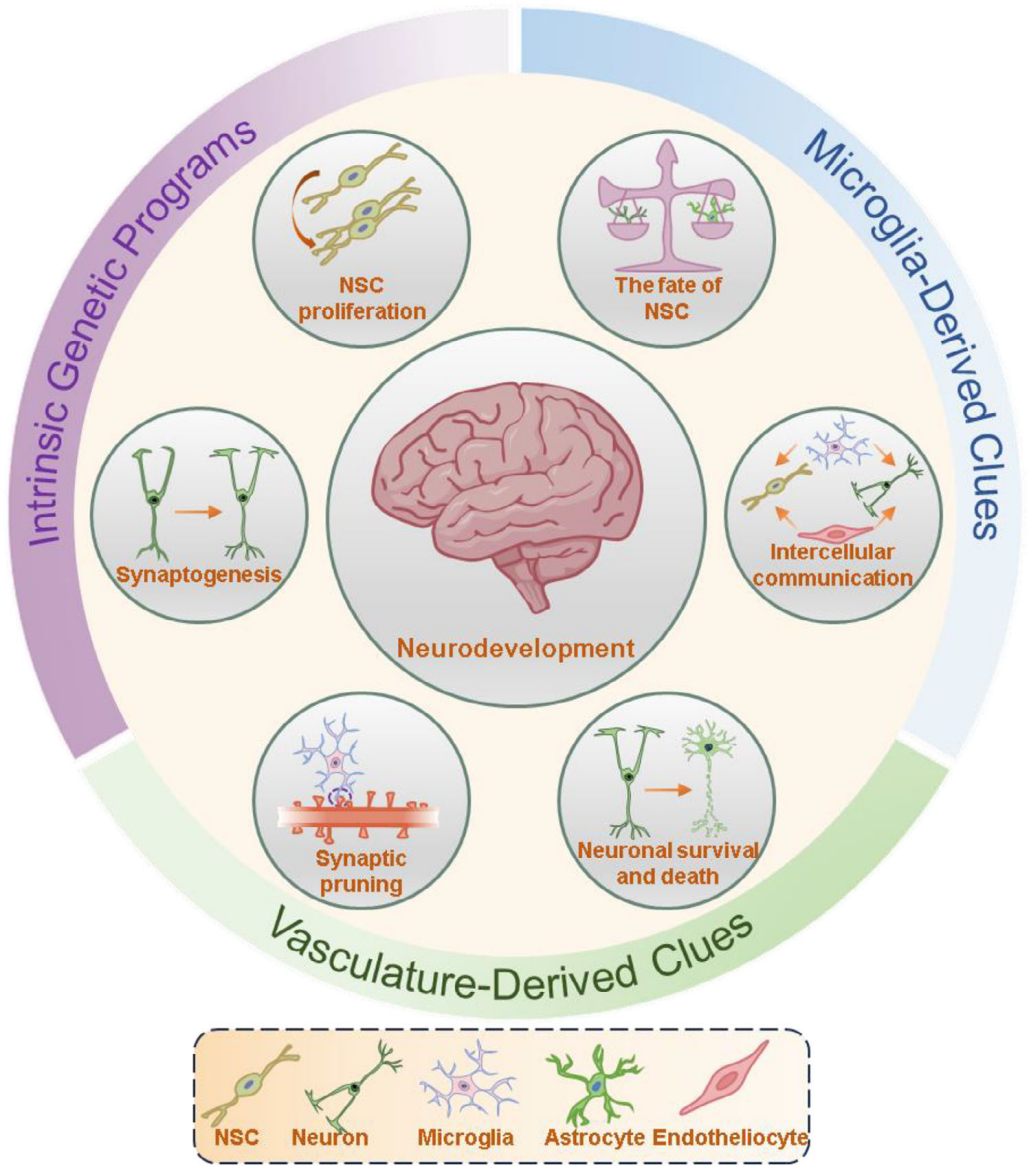
Overview of the regulation of intrinsic and extrinsic signals during neurodevelopment. Intrinsic genetic signals from NSCs, in conjunction with cues from microglia and blood vessels, collaboratively regulate the proliferation of NSCs, their fate determination, synaptogenesis, synaptic pruning, neuronal survival, and death, as well as communication between neural cells. Key cellular contributors to these processes include NSCs, neurons, microglia, astrocytes, and endotheliocytes.

The following sections will discuss the primary intrinsic and extrinsic regulatory factors of neural development in detail, including epigenetic modifications, vascular regulation of neurogenesis, and the role of microglia, as illustrated in **Figure** **1**. Additionally, the review will introduce key methodologies for studying neural development, such as single‐cell sequencing and gene knockout mouse models. Finally, it will summarize major advances in the field, analyze current challenges, and offer perspectives on future research directions.

## 2 Embryonic Neurogenesis

Neurogenesis in the mouse neocortex begins at embryonic day 10.5 (E10.5),^[^ ^1^ ^]^ while for humans, cortical development begins at the 7th week of pregnancy.^[^ ^2^ ^]^ At this time, the neuroepithelium located in the neural tube or neural plate begins to divide and produce radial glial cells (RGs).^[^ ^3^ ^]^ Radial glial cells are neural progenitor cells (NPCs) whose fate is more restricted than that of neuroepithelium. The differentiation from neuroepithelium to RGs depends on fibroblast growth factor 10.^[^ ^4^ ^]^ These newly formed RGs stack together to form the primitive region of neurogenesis, which is called the ventricular zone (VZ). RGs continue to divide and differentiate, generating intermediate progenitor cells (IPs) with lower self‐renewal ability and more restricted fate. These IPs construct new regions of neurogenesis called subventricular zone (SVZ). In the dorsal telencephalon of mice, the difference between IPs and apical radial glial cells (aRGs) is that IP expresses *Tbr2*, while aRG expresses Pax6.^[^ ^5^ ^]^ Next, aRGs and IPs generate neurons and form a functional neocortex. Compared to the murine neocortex, the primate cortex harbors an outer subventricular zone (oSVZ) containing intermediate progenitors, postmitotic neurons, and a substantial population of outer radial glia (oRG). These specialized oRGs are thought to underlie the evolutionary expansion of the human cerebral cortex and the formation of cortical gyri.^[^ ^6^ ^]^

### 2.1 Intrinsic Factors Affecting Neurogenesis

#### 2.1.1 DNA, RNA and Histone modification

Epigenetic modifications drive neurogenesis, with processes such as DNA methylation and histone acetylation ensuring the activation of neurogenesis‐related genes like *Sox2* and *NeuroD1* at specific developmental stages.

##### DNA Methylation

**Figure 2:**
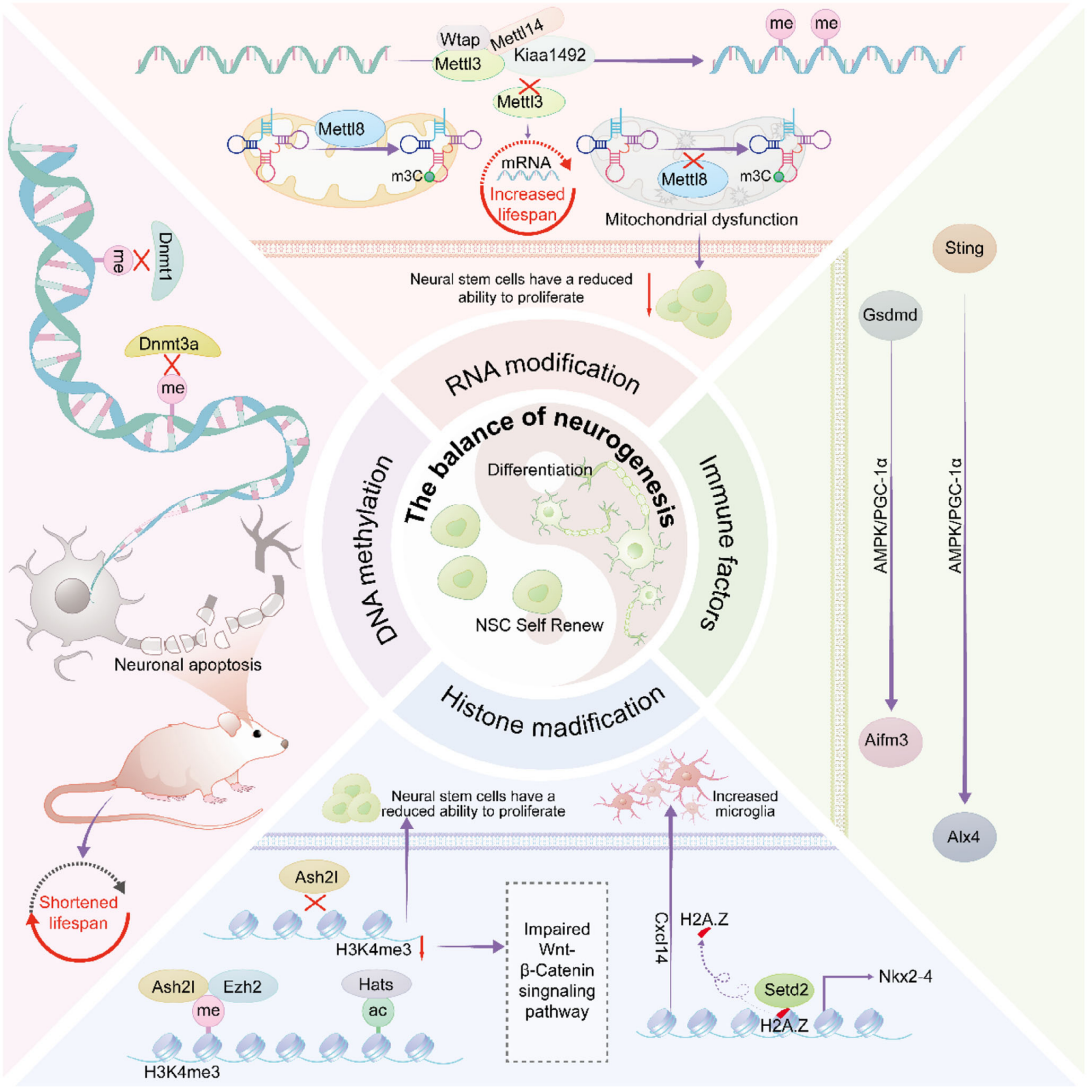
Overview of the regulation of NSC proliferation and differentiation by key genes during neurodevelopment. The circle in the center represents that the key to neural development lies in the balance between the proliferation and differentiation of NSCs. The remaining three sections systematically demonstrate the impacts of epigenetic modifications at the DNA, RNA, and histone levels on mouse brain development. DNMT family member deficiency blocks DNA methylation, leading to neuronal death and lifespan reduction in mice. Histone‐modifying enzymes *Ezh2* and Hats respectively catalyze histone methylation and acetylation, with their knockout causing abnormal NSC proliferation. Specifically, the deletion of histone variant H2A.Z in NSCs upregulates CXCL4 secretion, thereby perturbing microglial function. METTL8‐mediated mitochondrial tRNA modification maintains energy metabolism critical for NSC proliferation/differentiation, as *Mettl8*‐deficient mice exhibit impaired bioenergetics and perturbed cell fate decisions. Meanwhile, METTL3 promotes mRNA degradation through methylation, a process essential for NSC differentiation progression. The final section shows that during NSC proliferation, immune‐related genes *Gsdmd* and *Sting* are activated, primarily due to DNA damage, and this activation influences neurodevelopmental processes.

DNA methylation occurs on the fifth carbon atom of cytosine. This step is catalyzed by DNA methyltransferase. Dnmt1 mainly replicates the original methylation pattern during DNA replication, while Dnmt3b and Dnmt3a can generate new methylation regions. *Dnmt1* and *Dnmt3a* are expressed in NPCs during mouse embryonic development and neurons during late development and continue to be expressed throughout the brain after birth. *Dnmt3b* is only expressed from E10.5 to E15.5.^[^ ^7^ ^]^ DNA methylation is vital for brain development. *Dnmt1* deficiency in precursor cells in vivo leads to hypomethylation of mutated animal brain DNA and neonatal death. Surviving mice exhibit significant neuronal death after birth. Meanwhile, *Dnmt1* mutation leads to the failure of the development of the cortex and lost thalamocortical long‐term potentiation was observed in *Dnmt1* conditional mutant mice.^[^ ^8^ ^]^ The shortage of Dnmt3a in NSCs during embryogenesis leads to shortened lifespan, as well as reduced numbers of hypoglossal motor neurons.^[^ ^9^ ^]^ The main abnormalities caused by DNA methyltransferase defects in mice are summarized in **Table** **1** and **Figure** **2**.

**Table 1:** Neurodevelopmental defects and key gene deletions.

Target Gene	Tool Mice	Results	Refs.
*Dnmt1*	*Emx1* Cre	Somatosensory barrel cortex dysplasia. Lost thalamocortical long‐term potentiation.	[8b]
*Dnmt1*	*Nestin* Cre	Hypomethylation in brain. Neuronal death↑. Neonatal death↑.	[8a]
*Dnmt3a*	*Nestin* Cre	Hypoglossal motor neurons↓.	[9]
*Ash2l*	*D6* Cre	The proliferation ability of NSCs↓.	[21]
*Ezh2*	*Nestin* Cre	Hippocampal neurogenesis↓. Memory impairment.	[23]
*Kat8*	*Emx1* Cre	Apoptosis↑, Neurons↓.	[25]
H2A.Z.1	*Nestin* Cre	NPCs↑, neuronal↓ Abnormal dendrites.	[47]
H2A.Z.1	*hGFAP* Cre	Gliogenesis↓.	[29]
H2A.Z.2	*Nestin* Cre	Number of Microglia↑.	[30]
H2A.Z.2	*Cx3cr1* Cre	No abnormal phenotype in microglia.	[30]
*Mettl3*	*Nestin* Cre	Apoptosis of CGCs↑.	[11]
*Mettl3*	*Emx1* Cre	Neuron progenitor↓, Neuron↓.	[12]
*Mettl3*	*Six3* Cre	Retina abnormalities.	[13]
*Mettl14*	*Nestin* Cre	Delayed cortical neurogenesis. Number of astrocytes↓.	[14]
*Mettl14*	*Nestin* Cre	NSC proliferation↑, NSC differentiation↑,	[15]
*Mettl5*	Whole body knockout	Intellectual developmental defects.	[16]
*Mettl8*	*Nestin* Cre	Differentiation↑. NSC maintenance↑.	[17]
*Pd1*	*Nestin* Cre	NSC proliferation↑.	[48]
*Sting*	*Nestin* Cre	NSC proliferation↑.	[34]
*Gsdmd*	*Nestin* Cre	NSC proliferation↑.	[35]

In summary, abnormal DNA methylation during neurogenesis can lead to inhibition of neurogenesis. This may be related to abnormal gene expression associated with neurogenesis.

##### RNA Modification

There are over 170 modifications that occur on RNA. RNA modifications can occur on various types of transcripts. For example, N⁶‐methyladenosine (m⁶A) often occurs in mRNA, 5‐methylcytosine (m⁵C) can be found in long non‐coding RNA (lncRNA), and 3‐methylcytosine (m^3^C) is present in mitochondrial tRNAs.^[^ ^10^ ^]^ N6 methyladenosine (m6A) RNA methylation plays an important role in various cellular activities. The production of m6A RNA requires a methyltransferase complex, in which *Mettl3* plays an important role. The specific knockout of *Mettl3* in the mouse brain leads to developmental abnormalities in the cerebellum, caused by a sharp increase in apoptosis of cerebellar granule cells (CGCs) in the newly formed extracellular granule layer of the cerebellum. *Mettl3* depletion‐induced m6A modification deletion results in prolonged RNA half‐life, leading to transcriptional gene expression dysregulation and premature CGC death.^[^ ^11^ ^]^ Knocking out *Mettl3* in the cortex leads to translation changes of key genes in cortical NSCs, resulting in reduced production of Sox2 or Tbr2 positive NPCs and Tbr1 or Satb2 positive neurons.^[^ ^12^ ^]^ The lack of *Mettl3* can also impair the development of the retina manifested as disturbances in late‐stage retinogenesis.^[^ ^13^ ^]^ In addition to Mettl3, the key component of Mettl14 methyltransferase complex, specific knockout of *Mettl14* in NSCs results in a prolonged cell cycle of NSCs and neurogenesis extending into the postnatal stage. Knockdown of *Mettl3* also resulted in similar outcomes. In the absence of Mettl14, the expression of neuronal proteins can be detected in a large number of NPCs located in SVZ, indicating that the transcriptome mechanism plays a key role in hindering premature gene expression in neurogenesis.^[^ ^14^ ^]^ Y Wang et.al have also shown that the absence of *Mettl14* can result in a significant decrease in the premature differentiation of NSCs.^[^ ^15^ ^]^ In summary, m6A modification of mRNA has a significant role in cortex development, mainly by affecting the stability of RNA related to differentiation and proliferation in NPCs.

Besides mRNA methylation, rRNA methylation modification also plays a significant role in brain development. Mutations in the methyltransferase *Mettl5* lead to intellectual disability and microcephaly in patients. Neurodevelopmental abnormalities and intellectual deficits have been found in mice with *Mettl5* mutations.^[^ ^16^ ^]^ In the development of the brain cortex, research on m^3^C (3‐methylcytosine) modification is not very sufficient compared to m6A modification. Mettl8 exists in the mitochondria of NPCs and installs m^3^C on the tRNA^Thr/Ser (UCN)^ of mitochondria; conditionally knocking out *Mettl8* in NPCs leads to abnormal neural development, manifested in defects in embryonic cortical NSC maintenance. Mechanistically, Mettl8‐mediated tRNA modifications promote mitochondrial protein expression in neural stem cells. Loss of Mettl8 leads to impaired mitochondrial function. The use of pira, a compound that enhances mitochondrial function, can rescue neurodevelopmental defects caused by *Mett8* deficiency.^[^ ^17^ ^]^ The main abnormalities caused by RNA‐modifying enzyme defects in mice are summarized in Table 1 and Figure 2.

Although there is little research on the function of tRNA in neural development, considering the important role of tRNA modification in tumor cell proliferation and invasion, for example, a modification of tRNA, m^5^C, is important for the invasion of tumor cells. m^5^C modification in tRNAs increases protein synthesis in mitochondria. This helps cancer cells generate more energy to supply the consumption during invasion and metastasis.^[^ ^18^ ^]^ In the process of neurogenesis, NSCs have some similar energy production mechanisms to tumor cells through glycolysis. During the differentiation process of NPCs, there is a metabolic transition from glycolysis to aerobic oxidation. This metabolic transition is closely related to the maintenance of NSC proliferation ability and the generation of cerebral blood vessels.^[^ ^19^ ^]^ However, how mitochondrial tRNA modifications in NSCs affect brain development by regulating mitochondrial energy metabolism is not fully understood.

##### Histone Modifications and Variants

Different histone methylation sites are associated with activation or inhibition of gene transcription. H3K4 methylation is related to gene transcription activation. For example, Mll1 recognizes and binds to the promoter region or distal enhancer of the Dlx2 gene through its C‐terminal SET domain, catalyzes H3K4 methylation, and thereby induces *Dlx2* expression. If Mll1 is deleted, the histone modification at Dlx2 is replaced by H3K27 methylation, leading to repression of Dlx2 expression.^[^ ^20^ ^]^ *Ash2l* is essential for maintaining the production and localization of NPCs and projection neurons during the development of the neocortex. *Ash2l* deficiency can impair the trimethylation of H3K4 and the transcription mechanism of Wnt‐β‐Catenin signaling specificity, thereby suppressing the proliferative ability of NPCs in the late stage of brain development.^[^ ^21^ ^]^ Conversely, H3K27 methylation is related to gene transcription deactivation, PRC2‐catalyzed H3K27 methylation reduces Ngn1 expression, and Ngn1 promotes the conversion of NSCs into neurons. Inhibition of Ngn1 expression leads to reduced neurogenesis and premature gliogenesis.^[^ ^22^ ^]^ EZH2 is a subunit of PRC2, expressed in progenitor cells and mature neurons. *Ezh2* controls NSCs proliferation by inhibiting Pten and inducing AKT mTOR activation.^[^ ^23^ ^]^

Histone acetylation is commonly considered a regulatory mechanism of gene transcription. Histone acetylation happens in the lysine residues of H3 or H4 and is catalyzed by histone acetyltransferases and histone deacetylases.^[^ ^24^ ^]^ The specific knockout of *Kat8* in the developing brain of mice results in developmental defects in the neocortex and hippocampus, resulting in fewer, lower density, and increased apoptosis neurons in mutant mice; Intellectual disability, autism spectrum disorder (ASD), and other abnormalities exist in patient with *Kat8* mutation.^[^ ^25^ ^]^

Canonical histones are synthesized during DNA replication, whereas histone variants are continuously expressed in a replication‐independent manner within the cell. They can replace canonical histones in a process facilitated by histone chaperones. This replacement affects chromatin structure, thereby influencing gene expression.^[^ ^26^ ^]^ Histone variants are also involved in the process of neural development. Histone variants include H2A.X, H2A.Bbd, macroH2A, H2A.Z, CENP‐A, H3.X, H3.Y, H3.1t, H3.5, and H3.3.^[^ ^27^ ^]^ The absence of H2A.Z leads to increased proliferative ability of NPCs but decreased differentiation. In addition, mice that knock out the H2A.Z gene exhibit abnormal dendrites during cortex development. In addition, H2A.Z conditional knock‐out mice show a range of behavioral abnormalities. Mechanistically, the loss of H2A.Z significantly impacts Nkx2‐4 expression.^[^ ^28^ ^]^ Histone variants H2A.Z include H2A.Z.2 and H2A.Z.1. H2A.Z.1, which are essential for maintaining the multipotency of NSCs.^[^ ^26a^ ^]^ Deleting H2A.Z.1 results in reduced differentiation of glial cells. Mechanistically, H2A.Z.1 controls the modification of H3K56ac at the promoter of folate receptor 1. This directly results in a disturbance in the expression level of folate receptor 1. The changes in folate receptor 1 expression directly affect the JAK‐STAT signaling pathway, which is necessary for the development of glial cells.^[^ ^29^ ^]^ Knocking out H2A.Z.2 in NSCs resulted in increased microglia number during embryonic development. In addition, if H2A.Z.2 is deleted in microglia, the number and morphology of microglia are not affected. Mechanistically, the lack of H2A.Z.2 in NSCs promotes Cxcl14 production. Cxcl14 further results in an increase in the amount of microglia.^[^ ^30^ ^]^ Histone variant H3.3 is necessary for the proliferative ability of NPCs. Knocking down H3.3 results in a decrease in the number of Pax6‐positive NSCs, causing them to differentiate into neurons in advance. Mechanistically, H3.3 directly interacts with acetyltransferase Mof, leading to an increase in H4K16ac and a downregulation of the transcription regulatory factor Gli1 related to neurogenesis. Additionally, ChIP‐qPCR analysis revealed that H3.3, H4, and H4K16ac bind to the *Gli1* promoter region. This interaction specifically modulates MOF activity at this locus. By contrast, H3 or other histone isoforms do not exhibit similar effects.^[^ ^31^ ^]^ Recent studies have revealed that loss of H3.3 leads to downregulation of neurodevelopmental genes and perturbation of post‐translational histone modifications.^[^ ^32^ ^]^ In humans, individuals harboring germline H3.3G34R and H3.3G34V mutations exhibit severe cerebellar malformation and neurodevelopmental delay. Studies using corresponding murine models carrying these mutations revealed that the G34R substitution reduces H3K36me2 levels, impairs the recruitment of DNMT3A, and thereby alters DNA methylation patterns. These epigenetic perturbations promote the expression of complement and other immune‐related genes while suppressing neuronal gene transcription, ultimately disrupting neurodevelopmental programs.^[^ ^33^ ^]^

In summary, histone modifications influence chromatin state by altering histone charge and structure, thereby regulating gene transcription. Histone variants, conversely, replace canonical histones to change nucleosome stability, providing a structural basis for the formation of functional chromatin domains and thus affecting gene expression. Additionally, the research on histone modification mainly focuses on histone acetylation and methylation, but other modifications may also be involved in neurogenesis. The role of histone crotonylation, glycosylation, phosphorylation, succinylation, and ubiquitination are still not fully understood.

### 2.2 Inflammation‐Related Factors on Brain Development

#### 2.2.1 NSCs Proliferation Can Activate Immune Pathways

Endogenous stress caused by cell proliferation can activate many immune‐pathway‐related genes. These key molecules in the immune system, such as *Sting* and *Gsdmd*, have been found to play important roles in neurogenesis. Sting, as a protein that triggers an immune response to DNA, is detected in NSCs during brain development. In the early stage of cortical development, there is significant DNA damage in NSCs, and the expression of Sting is related to DNA damage. In *Sting* brain‐specific knockout fetal mice, there is abnormal distribution of neurons, including a significant increase in proliferating cells, a decrease in differentiated cells, and a certain degree of disorder in the morphology of neurons in the later stages. Sting insufficient mice exhibit autism‐like social behavior abnormalities in adulthood. The research team found through RNA‐Seq analysis of *Sting* deficient cortical cells that *Alx4* was significantly downregulated. Overexpression of *Alx4* can partially restore the abnormalities caused by Sting deficiency, and Sting can affect the expression of Alx4 by regulating the phosphorylation of NF‐κB.^[^ ^34^ ^]^ Hongyan et al. observed pyroptosis in cerebral cortex development. *Gsdmd* deficiency leads to excessive accumulation of DNA damage in NPCs throughout development. *Gsdmd* deficiency promotes the proliferation of NPCs and inhibits neuronal differentiation, resulting in autism‐like behavior in adult mice. Mechanistically, *Gsdmd* targets *Aifm3* by regulating the AMPK/PGC‐1α pathway.^[^ ^35^ ^]^ The main abnormalities caused by immune factors shortage in mice are summarized in Table 1 and Figure 2.

Overall, the absence of key molecules in the immune system seems to always promote the proliferative ability of NSCs, and offspring often exhibit autism or anxiety‐like behavior. These molecules often do not directly act but rather affect the genes related to neural development through a series of signaling pathways.

#### 2.2.2 Maternal Immune Activation and Abnormal Brain Development

Maternal infection with the Zika virus during pregnancy can lead to microcephaly in newborns, as Zika virus can directly replicate in fetal NSCs and further cause abnormal neurogenesis.^[^ ^36^ ^]^ In addition to direct virus infection of NSCs causing neurodevelopmental defects, using Poly (I:C) to simulate maternal virus infection can also cause fetal neurodevelopmental defects. Offspring of mothers injected with Poly (I:C) exhibit impaired neuronal generation and deficits in social skills after birth.^[^ ^37^ ^]^ In addition to viral infections, maternal parasitic infections can also lead to anxiety‐like and autism‐like behavior after birth, which is caused by changes in the composition of T cells.^[^ ^38^ ^]^ In addition to viruses and parasites, LPS has been used to simulate maternal bacterial infection and further observe fetal neural development and behavior in adulthood. These abnormalities include a decline in the number of neurons, activation of microglia, anxiety, and social deficits in adulthood.^[^ ^39^ ^]^

In conclusion, the rate of neurodevelopmental disorders (NDDs), such as ASDs, has increased rapidly year by year, suggesting that changes in the environment of fetal development play an important role. Maternal immune activation is one aspect, but other maternal changes such as obesity, diabetes, etc. have not been thoroughly studied for the clear relationship between the occurrence of NDDs.

### 2.3 Single Cell Sequencing Technology in Neurogenesis

#### 2.3.1 What Makes the Human Brain Unique?

The difference between the human brain and mice is manifested in the expansion of the neocortex and the formation of gyrification. The oSVZ is believed to explain the expansion of the human neocortex and the unique cognitive abilities of humans.^[^ ^6^ ^]^ Jing Liu et al. conducted transcriptome analysis of human NPC subpopulations and identified TMEM14B as a marker for human OSVZ RGs. Expression of TMEM14B in mice can induce cortical thickening and cortical gyrification formation. These transgenic mice are accompanied by the expansion of SVZ and the emergence of oRG‐like NSCs. Mechanistically, TMEM14B increases the phosphorylation and nuclear translocation of IQGAP1 to drive neuronal progenitor cell proliferation.^[^ ^40^ ^]^ Jinyue Zhao et al. reported that SERPINA3 is specifically expressed in human brain development, but the homologous gene *Serpina3n* in mice is not expressed during brain development. Using gene knock‐in technology to transfer human SERPINA3 into mice, the proliferation ability of neural cells in mice increased and the number of upper‐layer neurons increased, resulting in the appearance of gyrification and stronger learning and memory abilities in adulthood.^[^ ^41^ ^]^ Wenwen Wang et al. found that human FOXM1 can also promote cortical folding and the proliferation ability of NSCs in mice.^[^ ^42^ ^]^

#### 2.3.2 Single Cell Sequencing Reveals New Insights into Brain Development

Combining high‐dimensional flow cytometry with single‐cell transcriptomics, the cortical part was dissected from the developing human brain during the second trimester of pregnancy and prepared into a single‐cell suspension. Non‐neural cells were removed after multiple labeling. Meanwhile, the fluorescence intensity and transcription profile of each cell were analyzed, and the data were subjected to clustering and dimensionality reduction analysis. As a result, multiple types of RGs were identified.^[^ ^43^ ^]^ The first single‐cell RNA sequencing study on the developing human prefrontal cortex was conducted by Suijuan Zhong et al, revealed that the production of IPCs has two key burst periods, one is ≈10 weeks of embryonic development, and these IPCs are mainly produced by RGs, while the other peak occurs ≈16 weeks of embryonic development, and these IPCs are produced by oRGs. It is through the formation of these two explosive periods of IPCs that a large number of neurons can be rapidly generated during cortical development, forming a structurally complex and functionally rich prefrontal cortex.^[^ ^44^ ^]^ Yanxin Li et al. found that different subtypes of RGs are distributed in different brain regions during early human development. Neural precursor cells can be further divided into five subtypes. There are significant differences in the dorsal and ventral distribution of glutamatergic neurons and GABAergic neuronal subtypes.^[^ ^45^ ^]^ By integrating single‐cell RNA sequencing and scATAC‐seq technologies, dynamic changes in gene expression can be observed. For instance, early‐activated motifs include *PAX6, SOX2*, and *ASCL1*, mid‐stage activation involves *EOMES*, *NF1A*, *NF1B*, and *NEUROD1*, while late‐stage activation is marked by *NEUROD2*, *BHLHE22*, and *MEF2C*. This indicates the temporal activation of DNA motifs during brain development.^[^ ^46^ ^]^

While single‐cell sequencing technologies have advanced our understanding of brain developmental landscapes, several inherent limitations persist. First, the acquisition of high‐quality human fetal brain samples remains challenging due to factors such as pharmacological interventions administered prior to pregnancy termination, which may introduce molecular artifacts, coupled with ethical constraints that restrict accessibility to late‐gestation specimens. Second, cell type ambiguity arises from overlapping marker gene expression profiles between neural progenitor subtypes, exemplified by the transcriptional similarity between RGs and IPs. Third, the evolutionary divergence in progenitor cell dynamics, particularly the lack of human‐specific radial glial subtypes such as oRG in rodent models, imposes fundamental limitations on translating findings from rodents to human neurodevelopmental processes.

## 3 Contribution of Microglia to Neural Development

**Figure 3:**
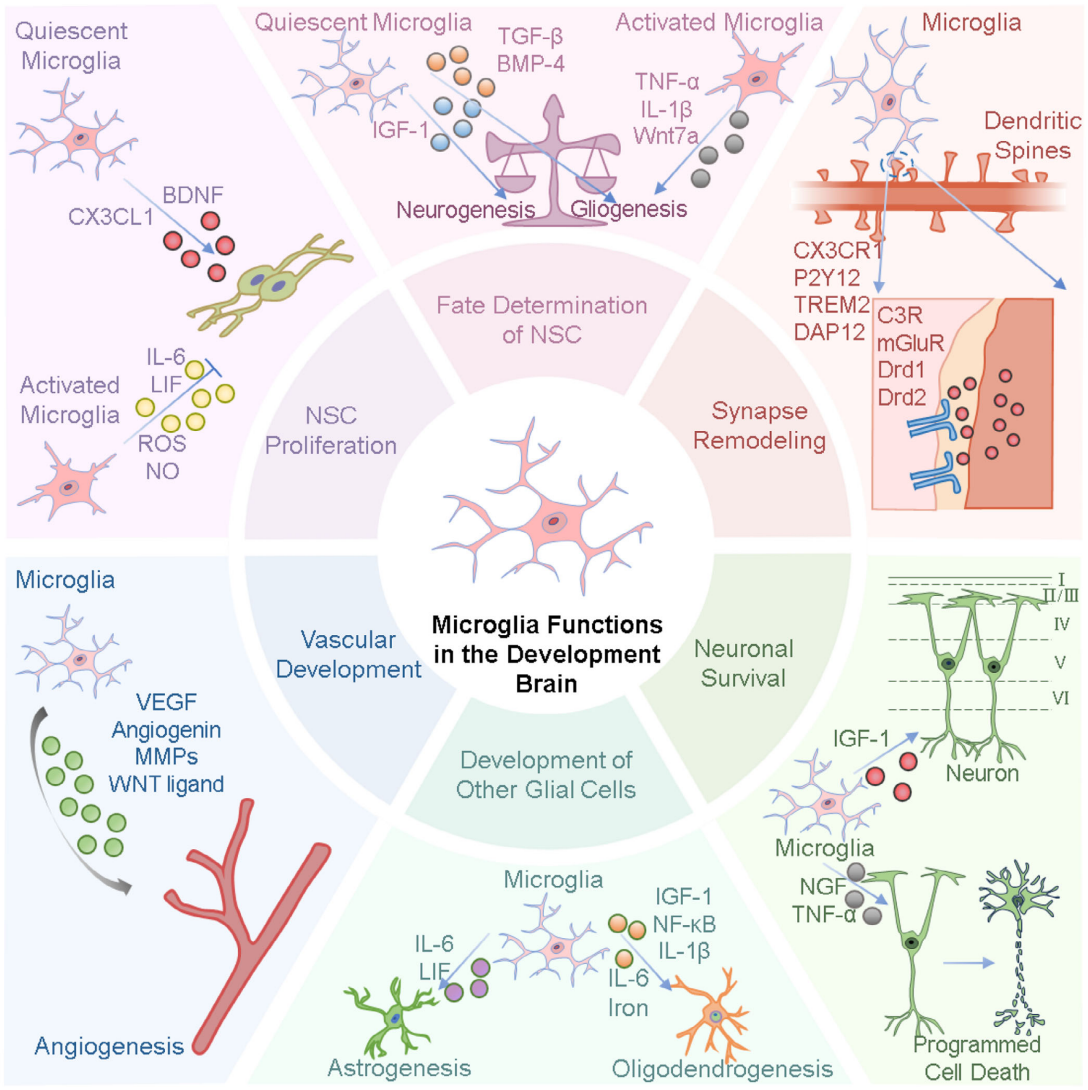
Role of microglia in neural development. This figure illustrates the diverse roles of microglia in neurodevelopment, highlighting their involvement in multiple key processes. Microglia exist in quiescent and activated states, responding to intrinsic and extrinsic signals to regulate neurogenesis and gliogenesis via factors such as TGF‐β, BMP‐4, IGF‐1, and inflammatory cytokines (e.g., TNF‐α, IL‐1β, Wnt7a). Microglia contribute to NSC proliferation by modulating signaling pathways involving IGF‐1, BDNF, IL‐6, LIF, ROS, and NO. Additionally, they participate in synapse remodeling through CX3CR1, P2Y12, TREM2, and other molecular mediators that influence dendritic spine formation and pruning. Microglia also regulate neuronal survival and programmed cell death, interacting with neurons via IGF‐1, NGF, and TNF‐α. Furthermore, they play a critical role in vascular development, promoting angiogenesis through VEGF, angiogenin, MMPs, and WNT ligands. Beyond neuronal interactions, microglia contribute to the development of other glial cells, including astrocytes and oligodendrocytes, by secreting IL‐6, LIF, iron, NF‐κB, and additional cytokines.

Neural development is a multifaceted process, governed by both genetic factors and environmental cues. Microglia, the resident immune cells within the central nervous system (CNS), play a pivotal role in regulating key neurodevelopmental processes, including NSC proliferation, fate determination, synaptic connectivity, and neuronal survival. Elucidating the specific mechanisms underlying these processes can provide essential theoretical insights for the prevention and intervention of NDDs. The subsequent sections delve into the distinct roles microglia play in various aspects of neural development, as shown in **Figure** **3**.

### 3.1 Overview of Microglia

Microglia are the resident immune cells of the CNS, which are characterized by their roles in maintaining immune homeostasis, clearing cellular debris, and dynamically interacting with neurons and glial cells. Their multifaceted functions in sustaining CNS equilibrium and mediating pathological responses have attracted extensive research interest.

#### 3.1.1 The Origin of Microglia

Microglia have been extensively studied for more than a century.^[^ ^49^ ^]^ In 1856, the German scientist Rudolph Virchow first identified what he termed “neuroglial cells”. Later, in 1919, Pío del Río‐Hortega identified a smaller type of glial cell that differed from both fibrous and protoplasmic astrocytes, which he termed microglia.^[^ ^50^ ^]^ Research in the 1980s and 1990s revealed that microglia in mice co‐express macrophage‐specific markers, including F4/80 and CD11b, while human microglia were found to express CD11b and Fcγ receptor I.^[^ ^51^ ^]^ The crucial study revealed that mice lacking the myeloid transcription factor PU.1 also lacked microglia,^[^ ^52^ ^]^ leading to the consensus that microglia originate from hematopoietic progenitors.

Although a consensus was reached regarding the myeloid origin of microglia, the precise identity of their progenitor cells remains contentious. This debate arises from the fact that microglia, despite being myeloid‐derived, are formed during embryonic development from two distinct sources of hematopoiesis: the yolk sac and the fetal liver. Consequently, researchers have focused on elucidating the specific lineage of microglia.^[^ ^53^ ^]^ Notably, Alliot's research demonstrated that microglia are detectable in the developing mouse brain as early as E8, shortly after the yolk sac forms, with their numbers progressively increasing until late gestation.^[^ ^54^ ^]^ Moreover, the application of various model organisms, including humans and zebrafish, has helped resolve the controversies surrounding microglial origins.^[^ ^55^ ^]^ Scientists have reached a consensus that mouse microglia originate from yolk sac macrophages present at E8.5. These macrophages migrate via the circulatory system into the developing brain, colonizing the neuroepithelium by E9.5. Following the establishment of the blood‐brain barrier (BBB) at E15.5, the interactions between the brain and fetal liver hematopoiesis are segregated, allowing for the expansion of microglia within the CNS as they finalize their network formation.^[^ ^56^ ^]^

#### 3.1.2 Distribution and Regional Heterogeneity of Microglia

Microglial colonization of the CNS begins in early embryonic development, originating from myeloid progenitor cells derived from the yolk sac. These progenitors migrate into the neural tube, where they differentiate into microglial precursors. Initially, these progenitor cells exhibit a relatively uniform distribution throughout the developing CNS. However, as brain maturation progresses, distinct patterns of microglial density emerge across various regions. By the time of birth, microglia display a heterogeneous distribution within the CNS, with particularly high densities in regions such as the hippocampus and olfactory bulb, while regions like the cerebellum and spinal cord exhibit lower densities. Research conducted by Yanxin Li et al. has revealed that the regional specialization of human immune‐related microglia is associated with the exit from a resting state, proposing the existence of microglial fate branching linked to neuronal gene enrichment in the human embryonic brain.^[^ ^45^ ^]^ This heterogeneity in microglial distribution remains consistent throughout the lifespan, indicating that microglia have adapted to local microenvironments and the specific needs of each brain region during CNS maturation. Several factors contribute to this regional specificity of microglia. During early development, the distribution of microglia is regulated by chemokine signals, such as CX3CL1 and CSF1, which guide their migration and colonization within neural tissue. The differential expression of these signals across various CNS regions may contribute to the observed variations in microglial density.^[^ ^57^ ^]^ Additionally, local neuronal activity and neurotrophic factors, such as brain‐derived neurotrophic factor (BDNF) and nerve growth factor (NGF), affect microglial proliferation and survival, contributing to the region‐specific variations seen in microglial populations.^[^ ^58^ ^]^

The density of microglia exhibits significant variability across different regions of the CNS, reflecting not only the unique demands of local neural circuits but also the distinct immunological surveillance requirements of various brain areas. In areas known for high synaptic plasticity, such as the hippocampus and cortex, microglial density is comparatively elevated, facilitating their involvement in synaptic remodeling and pruning. In these areas, microglia display highly branched morphologies with extensive protrusions, enabling them to monitor synaptic activity and respond to alterations in the local environment. Conversely, microglia in regions such as the cerebellum and spinal cord exhibit lower densities, which may indicate a reduced rate of synaptic turnover or a diminished need for immune surveillance. Moreover, the functions of microglia are modulated by region‐specific signals released by neurons and glial cells. For instance, astrocytes in the cerebral cortex secrete elevated levels of CX3CL1, a known chemokine that inhibits microglial activation, thereby maintaining a more quiescent state of microglia in that region. In contrast, microglia in areas like the hypothalamus may encounter lower concentrations of such inhibitory signals, leading to a more active or inflammatory baseline state.^[^ ^59^ ^]^

Microglia display significant regional variability in their distribution, density, and functional state throughout the CNS. Future investigations will focus on uncovering the molecular and cellular mechanisms that drive this regional specialization, providing crucial insights into the pathogenesis of neurological disorders and informing targeted therapeutic strategies for modulating microglial function in a region‐specific manner.

### 3.2 Role of Microglia in Neural Development

Neurogenesis in both the embryonic and adult brain is critically dependent on NSCs, which have the ability to self‐renew and differentiate into various neural lineages, such as neurons, astrocytes, and oligodendrocytes. The intricate regulation of NSC fate is governed by both cell‐intrinsic and extrinsic factors within their microenvironment. While microglia have traditionally been associated primarily with immune surveillance and neuroinflammation, recent evidence has positioned them as modulators of NSC proliferation and differentiation, thereby playing a significant role in neurogenesis.^[^ ^60^ ^]^ The following section provides a detailed overview of the roles played by microglia in neural development, along with the underlying molecular mechanisms involved.

#### 3.2.1 Impact on NSC Proliferation

The impact of microglia on the proliferation of NSCs is highly dependent on the homeostatic state of microglia. A study revealed that during the period from gestational week 8 (GW8) to GW16, proliferative and immune‐related microglia begin to establish a stable state by expressing homeostatic genes. However, the typical mature homeostatic gene SLC3A5 remains silenced during this period.^[^ ^61^ ^]^ By GW23, the immune‐related microglial subpopulations in the cerebrum, cerebellum, and diencephalon exhibit downregulation of quiescence‐related genes, while genes highly enriched in microglia associated with Alzheimer's disease, such as those related to activation and phagocytosis (e.g., APOE), are upregulated. This finding suggests that as embryonic development progresses, microglia undergo a transition out of their quiescent state.^[^ ^45^ ^]^ During early embryonic development, microglia preferentially colonize the VZ and SVZ. In their quiescent state, microglia secrete various trophic factors that promote NSC proliferation.^[^ ^60b^ ^]^ Benedetta Arnò et al. demonstrated that specific depletion of microglia leads to a reduction in the number of Tbr2+ cells in the SVZ at E13.5 during early neurogenesis in mice.^[^ ^62^ ^]^ Similarly, research by Yuki Hattori et al. showed that the administration of a CXCR4 antagonist, which inhibits microglial migration, results in a decreased abundance of Tbr2+ cells in the SVZ.^[^ ^63^ ^]^

In contrast, activated microglia markedly increase the release of pro‐inflammatory cytokines, which participate in the suppression of NSC proliferation by inducing apoptosis or causing cell cycle arrest.^[^ ^64^ ^]^ This activation of microglia is notably pronounced in the context of CNS injury or disease. Research conducted by Masaya Nakanishi et al. demonstrated that activated microglia release IL‐6 and leukemia inhibitory factor (LIF), which promote the differentiation of NSCs into astrocytes via the activation of the JAK/STAT and MAPK signaling pathways.^[^ ^65^ ^]^ Furthermore, reactive oxygen species (ROS) and nitric oxide (NO) derived from microglia exacerbate this proliferation‐related inhibition by inducing oxidative stress within the NSC microenvironment. Importantly, the extent of microglial activation and the duration of the inflammatory response are critical determinants of whether NSC proliferation is transiently suppressed or permanently impaired.

Research indicates that the phagocytic activity of microglia is crucial in regulating the population of NSCs. Cunningham et al. observed that microglia can phagocytose excess NPCs in the proliferative regions of the cerebral cortex in both rhesus monkeys and rats, without the need for these cells to undergo programmed cell death. This phagocytic activity persists throughout the neurogenesis process, peaking as neurogenesis approaches its conclusion.^[^ ^66^ ^]^ This finding suggests that microglia possess the ability to phagocytose viable cells, thereby maintaining the stem cell reservoir within neurogenic niches and finely tuning the proliferation rate of NSCs.

#### 3.2.2 Impact on the Fate Determination of NSC

The regulation of NSC fate by microglia represents a critical mechanism during CNS development. Recent investigations have elucidated the roles of microglia in both physiological and pathological contexts, revealing that microglia directly influence NSC fate through the secretion of various cytokines and growth factors. In the developing brain, microglia predominantly exhibit an anti‐inflammatory or resting phenotype, which facilitates NSC differentiation into neuronal lineages by secreting neurotrophic factors such as IGF‐1. This process not only promotes neurogenesis but also supports synapse formation and maturation.^[^ ^67^ ^]^ IGF‐1 is recognized as a key factor secreted by microglia, playing an essential role in both neuronal generation and the shaping of neural circuits.^[^ ^68^ ^]^ IGF‐1 binds to its receptor to activate various signaling pathways, especially the PI3K/AKT pathway, which promotes NSC survival and proliferation.^[^ ^69^ ^]^ Research by Sissel Ida Schmidt and colleagues has shown that microglia can influence the differentiation of human NSCs into dopaminergic neurons by releasing tumor necrosis factor‐alpha (TNF‐α), IL‐1β, and IGF‐1.^[^ ^70^ ^]^ Conversely, transforming growth factor‐beta (TGF‐β) secreted by microglia has been identified as a critical signal promoting the differentiation of NSCs into astrocytes.^[^ ^71^ ^]^ TGF‐β signaling is crucial not only for regulating glial cell generation during normal development but also for providing protection to the CNS under external stressors and neural injury through the induction of glial scar formation.^[^ ^72^ ^]^ However, this protective mechanism can have detrimental effects on neuronal function under certain circumstances. Overactive TGF‐β signaling may lead to excessive differentiation of NSCs into astrocytes, thereby limiting neuronal production and hindering regenerative potential.^[^ ^73^ ^]^ Moreover, Robert E. Gross and Weiwei Li were the first to identify BMP signaling as a regulator of astrogliogenesis and neuronal differentiation.^[^ ^74^ ^]^

The transition of microglial phenotype to a pro‐inflammatory state during neuroinflammation or CNS injury represents a significant regulatory factor in the fate of NSCs. When microglia adopt a pro‐inflammatory phenotype, they release high levels of inflammatory cytokines such as TNF‐α and IL‐1β. These cytokines negatively impact NSCs by not only inhibiting neuronal differentiation pathways but also by accelerating the commitment of NSCs to astrocytic and oligodendrocytic lineages, further promoting gliosis.^[^ ^75^ ^]^ This process is frequently associated with glial scar formation, which impedes regenerative repair within the CNS and is closely linked to the pathological progression of various neurodegenerative diseases, including Alzheimer's disease, Parkinson's disease, and traumatic brain injury.^[^ ^76^ ^]^ Additionally, findings from Pengxiang Zhu et al. reveal that co‐culture experiments involving microglia and NSCs show that microglia enhances the differentiation of NSCs into astrocytic lineages via the STAT3 signaling pathway.^[^ ^77^ ^]^

Microglia's dual role in regulating the fate of NSCs underscores their complex functions in both physiological and pathological contexts. While microglia contribute to normal brain development by supporting neurogenesis and modulating neural circuitry, their pro‐inflammatory phenotype can compromise neuroregenerative capabilities and exacerbate pathological processes under inflammatory or injury conditions. This phenomenon suggests the potential therapeutic value of targeting microglial phenotype transitions in neurodegenerative diseases. Future strategies aimed at modulating microglial activation states may offer novel approaches for promoting neural repair and regeneration.

#### 3.2.3 Contributions to Synaptogenesis and Synaptic Pruning

Synaptogenesis, maturation, and elimination are fundamental processes essential for the establishment and refinement of neural circuits, playing a critical role in the normal functioning of the CNS. While synapse formation predominantly occurs during development, synaptic remodeling persists throughout life, facilitating experience‐dependent plasticity and learning. The following section reviews the role of microglia in both synaptogenesis and synaptic pruning, highlighting the cellular and molecular mechanisms that regulate these processes.

Microglial processes continuously monitor the local environment and influence synaptogenesis by releasing neurotrophic factors and other signaling molecules at synaptic sites.^[^ ^78^ ^]^ Parkhurst et al. employed pharmacogenetic techniques to selectively deplete microglia in adult mice and those at three weeks postnatally, revealing that microglial depletion affected the formation and elimination of dendritic spines on layer 5 pyramidal neurons in the motor cortex, thereby disrupting spine turnover. Notably, the specific knockout of BDNF in microglia recapitulated the effects on new spine formation, indicating that this microglial‐derived neurotrophic factor promotes the development of new spines in the cortex.^[^ ^79^ ^]^ Subsequent in vivo imaging studies conducted in developing cortices further supported this role of microglia, demonstrating that interactions between microglia and dendritic spines during a critical developmental window enhance spine stability and growth, thereby facilitating synaptic maturation and structural refinement of excitatory synapses.^[^ ^80^ ^]^ Additionally, IGF‐1 secreted by microglia has been shown to promote synapse formation by regulating neuronal growth and synaptic connections.^[^ ^81^ ^]^ Furthermore, research suggests that the recruitment of microglia to mature synapses can influence the expression of synaptic receptors, with evidence that microglia modulate signaling factors associated with glutamate receptor activity, thereby facilitating the maturation of synaptic circuits.^[^ ^82^ ^]^

Microglia play a critical role not only in synapse formation but also in the selective pruning of synapses. This synaptic pruning occurs from early to late postnatal stages in mice and is vital for the remodeling of neuronal connections.^[^ ^83^ ^]^ Microglia are central to synaptic elimination, utilizing various molecular pathways to identify and engulf excess synapses.^[^ ^84^ ^]^ Previous studies have demonstrated that microglia exert physical surveillance over their microenvironment through ligand‐receptor interactions.^[^ ^85^ ^]^ Microglia display a range of neurotransmitter receptors, such as those for glutamate, gamma‐aminobutyric acid (GABA), and dopamine.^[^ ^86^ ^]^ Excessive release of glutamate activates glutamatergic receptors on microglia, triggering the production of reactive ROS and the release of ATP and cytokines, which in turn result in a reduction of dendritic spines and synaptic loss.^[^ ^87^ ^]^ Recent research has indicated that allosteric modulators of metabotropic glutamate receptor 5 can ameliorate synaptic over‐pruning in Alzheimer's disease models by preventing microglial phagocytosis of synapses tagged with complement component C1q.^[^ ^88^ ^]^ Additionally, microglia selectively prune inhibitory synapses through GABA signaling and GABAB receptor pathways,^[^ ^89^ ^]^ disruptions in this function during development may lead to attention‐deficit hyperactivity disorder in adulthood.^[^ ^90^ ^]^ Dopaminergic receptors, specifically *Drd2* in the amygdala and *Drd1* in the nucleus accumbens regulate synaptic pruning via mTOR and complement C3‐C3R signaling, respectively.^[^ ^91^ ^]^ This process is particularly active during critical periods of synaptic pruning in the developing brain. For example, in the visual cortex, microglial‐mediated synaptic pruning refines sensory maps and optimizes neural circuits. The roles of other neurotransmitters in microglia‐mediated synaptic pruning require further investigation. Neurons also secrete essential “find‐me” signaling molecules, such as chemokines and ATP. Neurons express the chemokine CX3CL1, which binds to its receptor CX3CR1 on microglia. CX3CR1‐deficient mice exhibit synaptic pruning deficits, leading to abnormal synaptic connectivity. ATP, secreted by neurons or astrocytes, binds to purinergic receptor P2Y12 on microglia, directing their processes to specific synapses and modulating synaptic plasticity.^[^ ^92^ ^]^ Furthermore, immune‐related receptors such as TREM2 and DAP12, expressed on microglia, have been implicated in regulating synaptic elimination and maintenance. Specifically, TREM2 contributes to microglial phagocytosis and is involved in the removal of synaptic debris during both development and disease.^[^ ^93^ ^]^

Microglia play a vital role in synapse formation and pruning, which is essential for the refinement of neural circuits during development and continuing into adulthood. Dysregulation of this process, characterized by excessive pruning or impaired synapse formation, is linked to various NDDs. A comprehensive understanding of the mechanisms underlying microglial‐synaptic interactions not only enhances our knowledge of neurodevelopmental processes but also reveals potential therapeutic targets for modulating synaptic connectivity in pathological contexts.

#### 3.2.4 Contribution to Neuronal Survival and Death

Microglia are crucial for enhancing neuronal survival during development. Ueno et al. demonstrated that the deletion of the CX3CR1 receptor in the microglia of mice led to a significant increase in apoptotic neurons within layer V of the postnatal cortical tissue. This increase in apoptosis was primarily attributed to a reduction in the secretion of IGF‐1 by the mutated microglia.^[^ ^94^ ^]^ IGF‐1 is known to be a crucial neurotrophic factor for the survival of neuronal precursor cells.^[^ ^95^ ^]^ Recent studies have further highlighted the impact of microglial depletion during embryonic development on the density and distribution of cortical neurons postnatally.^[^ ^96^ ^]^ Moreover, microglia are capable of phagocytosing both apoptotic neurons and those under survival stress.^[^ ^97^ ^]^ As early as 1998, José María Frade and colleagues reported that NGF secreted by microglia induced programmed cell death in developing chick retinas.^[^ ^98^ ^]^ Subsequently, Frédéric Sedel and associates revealed that microglia in the mouse spinal cord contribute to motor neuron death through the release of TNF‐α.^[^ ^99^ ^]^ Thus, microglia serve a dual function in development, supporting neuronal survival while also regulating neuronal death.

### 3.3 Role of Microglia on Non‐Neuronal Cell Development

Various studies have demonstrated that microglia exert regulatory effects on neuronal cells and play significant roles in the development of glial cells and the vascular system.

#### 3.3.1 Impact on the Development of Other Glial Cells

The development of astrocytes and oligodendrocytes begins during embryogenesis and continues into the postnatal period.^[^ ^100^ ^]^ Their precursors arise from radial glial cells, which later differentiate into mature astrocytes or oligodendrocytes. Microglia are present in the CNS from early development and play a key role in regulating the specification, proliferation, and differentiation of astrocyte and oligodendrocyte lineages.

Microglia influence the development of astrocytes and oligodendrocytes primarily through the secretion of growth factors. In vitro studies have demonstrated that IL‐6 and LIF secreted by microglia promote the differentiation of NSCs into astrocytes.^[^ ^65^ ^]^ Similarly, other secreted factors present in microglial conditioned media, such as IGF‐1, NF‐κB, IL‐1β, and IL‐6, have been shown to enhance the survival and differentiation of oligodendrocyte precursor cells into mature oligodendrocytes.^[^ ^101^ ^]^ Furthermore, related research has indicated that the transcription factor BACH1 alters microglial metabolic homeostasis, subsequently regulating astrocyte differentiation through the JAK/STAT3 signaling pathway.^[^ ^96b^ ^]^ Although the specific metabolic modes utilized by microglia during embryonic development remain unclear, it is reasonable to postulate that changes in microglial metabolic patterns are closely linked to their regulatory functions in neural development. Additionally, microglia promote myelination by supplying oligodendrocytes with iron, an essential cofactor for myelination.^[^ ^102^ ^]^ These findings indicate that microglia can influence the survival, proliferation, and maturation of various cell types within the CNS.

#### 3.3.2 Impact on the Vascular Development

Vascular development in the CNS is a tightly regulated process, with increasing evidence indicating that microglia play a key role in the formation of CNS blood vessels. Research has demonstrated that microglia infiltrate the CNS before vascular development, especially during retinal development, where they are closely associated with the invasion of blood vessels.^[^ ^103^ ^]^ In vivo studies using rodent models, along with in vitro studies involving microglial depletion, further underscore the necessity of microglia for the formation of vascular branches in the retina and hindbrain.^[^ ^104^ ^]^ Microglia stimulate endothelial cell proliferation and promote neovascularization by releasing angiogenic factors like vascular endothelial growth factor (VEGF) and angiopoietin.^[^ ^105^ ^]^ Microglia have also been shown to secrete matrix metalloproteinases (MMPs), particularly MMP‐9, which degrades extracellular matrix components essential for the remodeling required for angiogenesis.^[^ ^106^ ^]^ On the other hand, some studies have indicated contrasting effects. For instance, the depletion of microglia or the absence of WNT ligand transport proteins in microglia are associated with increased vascular branching in ex vivo retinal models.^[^ ^107^ ^]^ Future research is critical to fully understand the mechanisms through which microglia regulate angiogenesis. The context‐dependent activity of microglia emphasizes their dual role in either supporting or compromising vascular integrity, especially in reaction to neuroinflammatory signals. Understanding the molecular pathways through which microglia regulate vascular development is essential for elucidating their contributions to CNS pathology and for developing therapeutic strategies targeting microglial dysfunction in vascular diseases.

## 4 The Relationship Between Vasculature and the Nervous System During Development

In mice, the vascularization of the CNS begins ≈E8.5, with the formation of the perineural vascular plexus (PNVP) surrounding the neural tube.^[^ ^108^ ^]^ As endothelial cells continue to migrate and expand within the CNS, the development of cerebral vasculature gradually unfolds. The initially formed PNVP lays the foundation for subsequent vascular branching and extension. These endothelial cells progressively extend into the neural tube through a process called vascular sprouting.^[^ ^109^ ^]^ Guided by tip cells, vascular sprouts penetrate the outer layer of the neural tube and grow inward, driven by endothelial cell proliferation to form new vasculature.^[^ ^110^ ^]^ Tip cells, located at the leading edge of vascular sprouts, sense chemical signals in the external environment, such as VEGF and FGF, through filopodia, thereby directing the extension of new blood vessels. Meanwhile, stalk cells located behind tip cells are responsible for lumen formation, elongation, and branching.^[^ ^111^ ^]^

During brain development, vasculature and the nervous system exhibit a close collaborative relationship. The two are spatially and temporally intertwined, forming an interdependent developmental network. The vascular system plays supportive and guiding roles in nervous system development, not only providing nutrients and oxygen but also regulating neuronal proliferation, differentiation, and migration through the release of signaling molecules.^[^ ^112^ ^]^ First, the vascular network provides essential metabolic support for the developing nervous system. As the embryo grows rapidly, the demand for oxygen and nutrients increases sharply, and the establishment of the vascular system meets these needs. For instance, Jiao et al. demonstrated that the UCP2/ROS/ERK1/2 pathway in endothelial cells increases the expression of chymotrypsin‐1, enhancing angiotensin II (AngII) secretion in extra‐cranial endothelial cells.^[^ ^113^ ^]^ In highly active developmental tissues like the brain, the distribution and density of blood vessels often align closely with regions of NSC activity, highlighting the essential metabolic support provided by vasculature. Additionally, blood vessels serve as physical scaffolds for neuronal migration.^[^ ^114^ ^]^ During cortical formation, neurons migrate along the vascular network to specific locations, providing directional guidance for the construction of complex neural networks.

Second, various growth factors released by endothelial cells play critical roles in nervous system development. For example, VEGF not only regulates angiogenesis but also significantly influences neuronal survival, differentiation, and axon guidance.^[^ ^115^ ^]^ Other endothelial‐secreted factors, such as BDNF, similarly promote neural proliferation and differentiation. Through these signaling molecules, the vascular network achieves molecular‐level synchronization and coordination with nervous system development.^[^ ^116^ ^]^ Moreover, there is evidence of interaction and shared signaling pathways between the vascular and nervous systems during development. Pathways such as Notch, Wnt, and Netrin play roles in both systems, regulating cell fate determination and structural organization.^[^ ^117^ ^]^ By modulating the activity of blood vessels and NSCs, these pathways maintain balance and synchronization between the two systems during development.

In summary, the vascular system plays indispensable roles in nervous system development by providing nutrients and oxygen, releasing regulatory molecules, and serving as a physical scaffold. The close coordination between the two systems not only ensures normal neural development but also lays a foundation for future research into the molecular mechanisms underlying neurodevelopment. This interdependence highlights the co‐evolutionary mechanisms of vasculature and the nervous system in developmental biology, offering valuable insights into tissue engineering and regenerative medicine.

### 4.1 Interactions Between the Vascular and NSC Microenvironment

During neural development, interactions between the vascular system and the NSC microenvironment are accompanied by gradual changes in metabolic states, which play a critical role in regulating the fate of NSCs.^[^ ^118^ ^]^ In the early stages of embryonic development, the degree of vascularization in neural tissues is low, resulting in a hypoxic microenvironment with reduced oxygen levels.^[^ ^119^ ^]^ Under these conditions, NSCs primarily rely on anaerobic glycolysis for energy production. This mode of glycolytic energy metabolism is well‐suited for the early embryonic environment, providing sufficient ATP to support the rapid proliferation of NSCs.^[^ ^120^ ^]^ Moreover, the hypoxic environment activates the “quiescent” characteristics of NSCs, maintaining their high multipotency and stability, allowing them to better respond to the progression of vascularization and changes in oxygen availability later in development. This anaerobic metabolic state not only suits early embryonic conditions but also helps preserve the undifferentiated state of stem cells, enabling them to differentiate in response to environmental changes during subsequent developmental stages.^[^ ^121^ ^]^

As embryonic development progresses, blood vessels gradually infiltrate neural tissues, particularly in the neural tube and VZs, leading to a significant increase in oxygen and nutrient supply. NSCs begin to transition to aerobic metabolism, primarily relying on oxidative phosphorylation for energy production.^[^ ^122^ ^]^ This metabolic shift is crucial for the complex development of the nervous system, as oxidative phosphorylation is a highly efficient energy‐generating process capable of meeting the substantial energy demands required for the formation of intricate neural networks.^[^ ^123^ ^]^ In a high‐oxygen environment, reduced levels of metabolic by‐products stabilize the cellular state, supporting NSC differentiation and maturation. This allows NSCs to progressively develop into specific types of neurons or glial cells, aiding in the establishment of stable neural circuits.

The changes in metabolic states significantly influence NSC fate. Anaerobic metabolism in hypoxic environments supports NSC proliferation while maintaining their pluripotency and undifferentiated state. In contrast, under oxygen‐rich conditions following vascularization, NSCs become more responsive to differentiation signals, gradually transitioning into specific neurons or supportive cells. During this process, the metabolic shift not only alters energy supply but also modulates intracellular metabolic by‐products and redox states, further regulating gene expression and determining differentiation outcomes. Therefore, the changes in oxygen supply driven by vascular infiltration into neural tissues play a pivotal role in NSC metabolic transitions. Anaerobic metabolism in the early hypoxic environment ensures NSC proliferation, while enhanced aerobic metabolism in the oxygen‐rich environment after vascularization effectively guides NSC differentiation and maturation. This gradual metabolic transition underpins the precise regulation between the vascular system and the NSC microenvironment and is a key factor for normal neural development and the formation of complex neural functions.

Emerging evidence highlights the critical role of vascular abnormalities in neurodevelopmental disorders, where disrupted blood supply and angiogenesis impair brain development. In hypoxic‐ischemic encephalopathy (HIE), for instance, vascular insufficiency leads to chronic hypoxia, compromising NSC proliferation and cortical layering. Hypoxia disrupts metabolic support for NSCs, triggering premature differentiation or apoptosis, ultimately thinning the cortex and disrupting synaptic connectivity.^[^ ^124^ ^]^ Aberrant angiogenesis further exacerbates these deficits, as mispatterned vessels fail to meet the metabolic demands of developing neural circuits. Notably, vascular dysregulation is also implicated in ASD and schizophrenia. Postmortem and imaging studies in ASD reveal altered cerebral blood flow and tortuous microvasculature, correlating with regions of synaptic overgrowth and hyperconnectivity.^[^ ^125^ ^]^ Similarly, in schizophrenia, prenatal hypoxia and reduced cortical perfusion are linked to aberrant dendritic arborization and disrupted GABAergic interneuron migration.^[^ ^126^ ^]^ These findings underscore vascular dysfunction as a shared mechanism perturbing neurogenesis, circuit assembly, and functional network formation across neurodevelopmental conditions.

However, while extrinsic signaling pathways are known to play crucial roles in neural development, studies by Sally Temple's team demonstrated that NPCs exhibit cell‐autonomous regional specification, independent of extrinsic signals.^[^ ^127^ ^]^ In Drosophila, neuroblasts acquire distinct identities via lineage‐intrinsic programs rather than environmental cues.^[^ ^128^ ^]^ Similarly, vertebrate NPCs from different CNS regions maintain region‐specific gene expression even in uniform culture conditions, suggesting pre‐patterning at the stem cell level. For example, *Sox2* expression is epigenetically confined to telencephalic progenitors, whereas it is absent in spinal cord progenitors, further substantiating the concept of intrinsic fate commitment.^[^ ^129^ ^]^ In a complementary study, Denis Jabaudon's team demonstrated that apical progenitors retain temporal plasticity. They can revert to earlier molecular, electrophysiological, and neurogenic states when exposed to a younger microenvironment. This reprogramming is driven by their ability to sense dynamic WNT signaling changes in the extracellular environment.^[^ ^130^ ^]^

The behavior and fate determination of NSCs are co‐regulated by extrinsic metabolic/angiogenic signals and intrinsic cellular programs. The extrinsic microenvironment provides essential nutritional support and regulatory signals that influence NSC survival and proliferative states, while the intrinsic programs maintain their inherent developmental potential and regional identity to ensure fate determination stability. These two mechanisms work in concert, enabling NSCs to flexibly respond to environmental changes while preserving the precision of developmental execution, thereby coordinating normal nervous system development and functional maintenance.

### 4.2 Physical Support of NSCs by Vascular Scaffolds

During cortical development, the migration of inhibitory GABAergic interneurons is orchestrated by complex and finely regulated mechanisms, with the vascular system playing a crucial role not only as a physical scaffold but also as a source of key molecular cues.^[^ ^131^ ^]^ Unlike excitatory cortical neurons, which migrate radially along radial glial fibers, GABAergic interneurons originate in the ventral telencephalon, primarily within the medial, lateral, and caudal ganglionic eminences, as well as the preoptic area. These interneurons migrate tangentially over long distances to reach the developing neocortex, and their trajectories are closely associated with the brain's vascular network.

GABAergic interneurons navigate two main migratory streams: the superficial migratory stream (SMS) and the deep migratory stream (DMS). The SMS runs beneath the pial surface, whereas the DMS traverses the subplate and intermediate zone.^[^ ^132^ ^]^ Along these pathways, blood vessels provide essential guidance cues. Notably, endothelial cells from the pial and periventricular vascular networks exhibit distinct gene expression profiles and secrete different levels of GABA. High concentrations of GABA secreted by pial endothelial cells guide interneurons within the SMS, while lower concentrations released by periventricular endothelial cells influence migration within the DMS.^[^ ^133^ ^]^ GABAergic interneurons sense these gradients, modulating their migratory speed and direction accordingly to achieve precise spatial distribution.

In addition to acting as a diffusible guidance molecule, GABA activates GABAA receptors on migrating neurons, thereby regulating intracellular calcium dynamics and influencing cytoskeletal remodeling necessary for migration. Moreover, endothelial cells themselves express GABAA receptors, enabling GABA to promote angiogenesis via autocrine signaling.^[^ ^133^ ^]^ Consequently, disruption of endothelial GABA release impairs both vascular development and interneuron migration, highlighting the interdependence of these processes.

In summary, blood vessels not only provide structural support but also secrete critical molecular signals, such as GABA, to orchestrate the long‐distance tangential migration of GABAergic interneurons during cortical development. This vascular–neuronal interaction is fundamental for the establishment of proper cortical circuitry. Further investigation into the dynamic interplay between blood vessels and migrating neurons may offer new insights into the pathogenesis of neurodevelopmental disorders, including epilepsy and autism spectrum disorders.

### 4.3 Role of Vascular in Neural Development

#### 4.3.1 Effects on NSC Proliferation and Differentiation

During neural development, the vascular system not only supplies oxygen and nutrients to NSCs but also directly regulates their proliferation and differentiation through the release of various signaling molecules, such as VEGF, FGF, and Notch signaling molecules. Jiao et al. demonstrated that RNF20 in endothelial cells also indirectly promotes the proliferation and differentiation of NPCs by secreting CILP2.^[^ ^134^ ^]^ These molecules extend the proliferation window of NSCs while promoting their differentiation into specific neuronal subtypes at the appropriate time.

VEGF, a key factor secreted by vascular endothelial cells, plays a dual role in the NSC microenvironment: promoting angiogenesis while significantly influencing NSC proliferation during neural system development. Studies have shown that VEGF binds to VEGFR receptors on NSCs, activating downstream signaling pathways to maintain their undifferentiated state and prolong their proliferation.^[^ ^135^ ^]^ In this way, vascular‐derived VEGF provides sufficient cellular resources to support early neural development, playing a critical role in sustaining neurogenesis.

The Notch signaling pathway serves as a critical mediator in vascular‐NSC crosstalk during adult neurogenesis. Experimental evidence demonstrates that vascular contact‐induced Notch activation in adult NSCs suppresses their differentiation potential while promoting proliferation.^[^ ^136^ ^]^ While Notch signaling is known to play an important role in embryonic neurogenesis, its specific regulation by vascular contacts during embryonic stages requires further investigation. This contact‐dependent interaction highlights the spatial and signaling regulatory importance of blood vessels, allowing NSCs to maintain an appropriate proliferative state or initiate differentiation based on developmental needs.

In summary, blood vessels regulate NSC proliferation and differentiation through VEGF, FGF, and Notch signaling pathways at multiple levels. The role of vasculature in neural development extends beyond metabolic support, directly influencing NSC growth and fate decisions through these molecular mechanisms. These findings provide profound insights into the role of blood vessels in neural development and open new possibilities for interventions in neural regeneration and developmental abnormalities.

#### 4.3.2 Effects on Synapses

Vascular endothelial cells secrete VEGF, a critical factor in synapse regulation. VEGF not only promotes angiogenesis but also directly influences the formation and stabilization of neuronal synapses through binding to VEGF receptors (e.g., VEGFR2) on neurons.^[^ ^137^ ^]^ Upon binding to VEGFR2, VEGF activates downstream signaling pathways, including the PI3K/AKT and MAPK/ERK pathways, which are essential for promoting synaptic growth and enhancing synaptic stability.^[^ ^138^ ^]^ Carsten et al. found that VEGF signaling enhances neuronal growth cone activity and increases axonal branching at terminal ends, thereby promoting synapse formation and complexity.^[^ ^139^ ^]^ Through these mechanisms, VEGF signaling contributes to synaptogenesis during neural development and plays a role in brain plasticity, learning, and memory.

In addition, platelet‐derived growth factor (PDGF), released by the vascular system, significantly affects synaptic structure and stability. PDGF, through its receptor PDGFR on neurons, regulates cytoskeletal reorganization, impacting the structural stability of presynaptic neurons.^[^ ^140^ ^]^ PDGF activates the *Rho* family GTPases, particularly *Rac1* and *Cdc42*, which control the microfilament and microtubule structures at presynaptic terminals, providing essential support for synaptic connections.^[^ ^141^ ^]^ PDGF signaling also modulates calcium influx to enhance synaptic excitability.^[^ ^142^ ^]^ These molecular mechanisms position PDGF as a key regulator of synaptic plasticity, especially during synaptic remodeling or adaptive changes, ensuring that synapses can meet new functional demands.

BDNF is another vascular‐associated key factor crucial for synaptic maturation and functional regulation.^[^ ^143^ ^]^ By binding to its receptor TrkB, BDNF activates multiple downstream pathways, including PLCγ, PI3K, and MAPK, which promote dendritic spine formation and expansion in postsynaptic neurons, stabilizing and enhancing synaptic efficiency.^[^ ^144^ ^]^ Research by Konnerth et al. demonstrated that BDNF signaling enhances long‐term potentiation, a synaptic plasticity mechanism fundamental to learning and memory.^[^ ^145^ ^]^ BDNF is found at higher concentrations in regions adjacent to blood vessels, and through diffusion or active secretion, it influences the efficiency and stability of neuronal synaptic connections, enabling synapses to adapt to external stimuli and changes in neuronal activity.

Additionally, blood vessels secrete SDF‐1 (stromal cell‐derived factor 1, also known as CXCL12), which impacts synaptic repair and regeneration. SDF‐1 binds to its receptor CXCR4, activating the ERK and PI3K pathways to enhance synaptic growth and recovery. In particular, SDF‐1 effectively promotes synaptic regeneration following synaptic damage.^[^ ^146^ ^]^ This mechanism is especially crucial for post‐injury neural repair, as vascular‐derived SDF‐1 signaling enhances the regenerative capacity of damaged synapses, supporting neural system recovery.

In summary, the vascular system regulates synapse formation, maturation, and repair through the secretion of factors such as VEGF, PDGF, BDNF, and SDF‐1. These molecules activate downstream signaling pathways, including PI3K/AKT, MAPK/ERK, *Rho* GTPase, and PLCγ, precisely controlling dynamic changes and functional enhancement in both presynaptic and postsynaptic structures. The vascular system plays a multi‐level role in the molecular regulation of synapses, ensuring their stability and plasticity and providing a molecular foundation for neural system development, learning, and memory functions.

#### 4.3.3 Effects on Neurons

During the development of the nervous system, blood vessels not only provide essential metabolic support but also directly regulate neuronal growth, survival, differentiation, and migration through the secretion of various signaling molecules. In addition to common factors such as VEGF and FGF, vascular‐derived molecules like IGF, hepatocyte growth factor (HGF), stem cell factor (SCF), and pigment epithelium‐derived factor (PEDF) play critical roles, offering diverse mechanisms for the fine regulation of neural development.^[^ ^147^ ^]^

IGF‐1, a key growth factor secreted by vascular endothelial cells, supports neuronal growth, differentiation, and survival in multiple ways. Upon binding to IGF‐1R receptors on neurons, IGF‐1 activates the PI3K/AKT and MAPK/ERK signaling pathways, enhancing neuronal anti‐apoptotic capacity and promoting proliferation and differentiation.^[^ ^148^ ^]^ This function is especially critical during early development when neurons need to expand rapidly to construct the initial neural network. IGF‐1 also promotes axon elongation and dendritic branching, laying the groundwork for neural network complexity.^[^ ^149^ ^]^ Additionally, IGF‐1 works synergistically with other factors, such as VEGF and BDNF, to provide neurons with multifaceted support, enhancing their stability and adaptability in the developmental environment.^[^ ^150^ ^]^

HGF binds to its receptor c‐Met, activating multiple downstream pathways, including PI3K, MAPK, and STAT3, which are crucial for neuronal migration, differentiation, and axonal growth. HGF not only promotes neuronal survival but also acts as a chemoattractant, guiding neurons along specific paths to their target locations.^[^ ^151^ ^]^ By regulating cytoskeletal reorganization, HGF increases neuronal extension capacity, facilitating the spatial arrangement of neurons in complex neural networks.^[^ ^152^ ^]^ This chemoattractive effect is essential for the layered organization of the nervous system, ensuring precise spatial positioning of neurons.

SCF interacts with c‐Kit receptors on neurons, activating intracellular signaling pathways that support neuronal proliferation, anti‐apoptotic mechanisms, and migratory capabilities.^[^ ^153^ ^]^ The SCF/c‐Kit signaling pathway is particularly active during embryonic development, promoting the differentiation of NPCs into specific neuron types and aiding their localization in the developmental space.^[^ ^154^ ^]^ Additionally, SCF enhances neuronal migration and distribution by modulating cytoskeletal dynamics.^[^ ^155^ ^]^ SCF provides critical positional support for neurons during the initial formation of the neural network, ensuring proper hierarchical organization of the nervous system.

PEDF is an important molecule in the nervous system that exerts anti‐apoptotic and antioxidant effects. PEDF interacts with multiple receptors on neurons, activating anti‐apoptotic signaling pathways and protecting neurons from environmental stress, particularly under oxidative stress conditions.^[^ ^156^ ^]^ Moreover, PEDF plays a unique role in neural repair following injury, enhancing neuronal resilience to damage and promoting regeneration.^[^ ^157^ ^]^ PEDF's neuroprotective role makes it an essential support molecule for stabilizing neural network structures during later stages of neural development.

In summary, vascular‐derived molecules such as IGF‐1, HGF, SCF, and PEDF regulate neuronal growth, survival, migration, and differentiation through multiple mechanisms. The synergistic actions of these factors provide multilayered support for normal neural development, ensuring the structural and functional integrity of neural networks.

### 4.4 Role of Vascular on Non‐Neuronal Cell Development

#### 4.4.1 Influence on Astrocytes

During nervous system development, the proliferation, differentiation, and functional maturation of astrocytes are finely regulated by various signaling molecules. While astrocyte development depends on the metabolic support provided by the vascular system, specific molecular pathways also play critical roles in regulating astrocyte development. These molecules include BMP, cAMP response element‐binding protein (CREB), glial cell line‐derived neurotrophic factor (GDNF), and the transcription factor NFIA, which collectively establish a unique basis for astrocyte development and functionality.^[^ ^158^ ^]^

The BMP signaling pathway plays a pivotal role in determining astrocyte fate. During development, BMP binds to its receptor BMPR, activating the downstream SMAD pathway, which promotes the differentiation of NSCs into astrocytes.^[^ ^159^ ^]^ Nakashima et al. demonstrated that BMP signaling not only suppresses neuronal traits but also specifically enhances astrocyte characteristics, enabling them to develop mature morphology and functionality.^[^ ^160^ ^]^ This role of BMP provides a molecular foundation for astrocyte functional maturation, helping them support neurons and maintain homeostasis within the CNS.

CREB primarily influences astrocyte proliferation and anti‐apoptotic capacity by regulating gene expression. As a key transcription factor, CREB is activated by cAMP signaling, after which it translocates into the nucleus to regulate genes associated with cell proliferation and survival.^[^ ^161^ ^]^ During astrocyte maturation, CREB signaling helps these cells adapt to dynamic environments and modulate their morphology and distribution. CREB also plays a crucial role in the formation of astrocytic processes, enabling astrocytes to cover larger neuronal areas, closely interact with synapses, and maintain the stability of neural networks.^[^ ^162^ ^]^ Furthermore, CREB signaling regulates astrocytes’ responsiveness to neurotransmitters, allowing them to actively support neuronal activity and modulate synaptic plasticity.

GDNF is essential for astrocyte growth and functional stability. By binding to its receptors, RET and GFRα1, GDNF activates the PI3K/AKT and MAPK pathways, promoting astrocyte survival and antioxidative capacity.^[^ ^163^ ^]^ Iwaki et al. found that GDNF significantly regulates the distribution and extension of astrocytes, facilitating the growth of astrocytic processes and enabling them to cover broader neuronal network areas. This molecular mechanism is crucial for protecting neurons, ensuring that astrocytes provide stable support within the complex neural environment.^[^ ^164^ ^]^

The transcription factor NFIA holds a unique position in astrocyte fate determination and serves as a central regulator of astrocyte development.^[^ ^165^ ^]^ During embryonic development, NFIA directs NSCs toward astrocyte differentiation and regulates the expression of specific genes required for their complex morphology and function. NFIA not only promotes astrocyte differentiation but also ensures their proper distribution within neural networks. This ensures that astrocytes effectively support neuronal metabolism, contribute to the formation of the BBB, and maintain the homeostasis of the nervous system.^[^ ^166^ ^]^

In summary, the development of astrocytes is tightly regulated by BMP, CREB, GDNF, and NFIA through specific molecular mechanisms. These molecules modulate astrocyte proliferation, differentiation, migration, and functional stability via distinct signaling pathways. Such mechanisms endow astrocytes with unique abilities to collaborate with neurons, providing multi‐level support and protection to the nervous system. The critical roles of astrocytes in neural networks highlight their importance in CNS development and homeostasis, offering new perspectives for understanding the intricate regulation of astrocyte functions during neural development.

#### 4.4.2 Effects on Oligodendrocytes

During nervous system development, the vascular system supports oligodendrocyte development not only by providing metabolic support but also by secreting specific molecules that directly regulate their proliferation, differentiation, migration, and myelination processes. For example, endothelial STING promotes cholesterol synthesis by regulating FDFT1, thereby enhancing myelination. Other key molecules include IL‐33, endothelin‐1 (ET‐1), angiopoietin‐1 (Ang‐1), and thrombin, which collectively provide fine‐tuned regulation of oligodendrocyte development and function.^[^ ^167^ ^]^

IL‐33, a cytokine secreted by vascular endothelial cells, plays a crucial role in the differentiation and maturation of oligodendrocytes. IL‐33 binds to the ST2 receptor on oligodendrocytes, activating the downstream JAK/STAT signaling pathway, which promotes the differentiation of oligodendrocyte precursor cells into mature oligodendrocytes and enhances their myelination capabilities.^[^ ^168^ ^]^ Yang et al. found that IL‐33 significantly improves the anti‐inflammatory and anti‐apoptotic capacities of oligodendrocytes, enabling them to better adapt to environmental changes during myelination.^[^ ^169^ ^]^ The absence of IL‐33 leads to differentiation defects and incomplete myelination, highlighting its critical role in oligodendrocyte maturation.

ET‐1, another molecule secreted by vascular endothelial cells, primarily regulates the migration and anti‐apoptotic abilities of oligodendrocytes.^[^ ^170^ ^]^ By binding to ETB receptors on oligodendrocytes, ET‐1 modulates intracellular calcium channels, enhancing cell stability and ensuring their survival and function in specific regions.^[^ ^171^ ^]^ ET‐1 also facilitates cytoskeletal reorganization, helping oligodendrocytes distribute and extend across the nervous system.^[^ ^172^ ^]^ Additionally, ET‐1 plays a protective role in responding to neuroinflammation and repairing damage, supporting oligodendrocyte stability during myelination.^[^ ^173^ ^]^

*Ang‐1* plays a key role in the survival and differentiation of oligodendrocytes.^[^ ^174^ ^]^ By binding to the Tie2 receptor, *Ang‐1* activates the PI3K/AKT signaling pathway, enhancing the anti‐apoptotic capacity of oligodendrocytes and providing sustained developmental support.^[^ ^175^ ^]^ During CNS development, *Ang‐1* signaling helps oligodendrocytes adapt to metabolic and oxygen stress, allowing them to maintain normal functions even under hypoxic conditions.^[^ ^176^ ^]^ This molecule is critical for both the formation and maintenance of myelin, demonstrating that the vascular system supports oligodendrocytes not only through metabolic means but also by boosting their survival and functional stability via molecular signaling.

Thrombin, a vascular‐associated molecule, also plays a regulatory role in oligodendrocyte differentiation and myelination. By activating protease‐activated receptors‐1 on oligodendrocytes, thrombin promotes the expression of myelin‐related genes and accelerates the maturation process of oligodendrocytes.^[^ ^177^ ^]^ Thrombin signaling is particularly significant during the early stages of myelination, where it fine‐tunes intracellular signaling networks to enable rapid myelin formation. Moreover, thrombin enhances the repair capacity of oligodendrocytes in cases of neural damage, aiding in the regeneration of damaged myelin.^[^ ^178^ ^]^

In summary, the vascular system regulates the proliferation, differentiation, migration, and myelination of oligodendrocytes through the secretion of IL‐33, ET‐1, ANG‐1, and thrombin. Compared to traditional growth factors, these molecules reveal a more sophisticated regulatory mechanism by which the vascular system ensures that oligodendrocytes complete their maturation at the right time and place, forming healthy myelin. This, in turn, supports efficient signal conduction and functional stability in the CNS. These findings provide new perspectives for understanding myelination and the role of oligodendrocytes in the nervous system.

#### 4.4.3 Effects on Microglia

The vascular system plays a critical regulatory role in the development and function of microglia. Microglia, as the primary immune cells of the CNS, are involved in clearing damaged or dysfunctional cells, repairing neural networks, and modulating inflammatory responses. By secreting specific signaling molecules such as chemokines, cytokines, and growth factors, the vascular system influences microglial proliferation, differentiation, migration, and distribution within the CNS.^[^ ^179^ ^]^

First, CXCL12 (also known as SDF‐1), a chemokine secreted by vascular endothelial cells, is essential for microglial migration and positioning. CXCL12 binds to the CXCR4 receptor on microglia, creating a chemotactic gradient that guides their migration along vascular pathways to target regions.^[^ ^180^ ^]^ This chemotactic guidance ensures that microglia are accurately positioned in areas requiring immune support during early development and neural repair. The CXCL12/CXCR4 signaling pathway is crucial for the spatial distribution of microglia, ensuring adequate immune cell coverage across the CNS to maintain tissue homeostasis.

Vascular endothelial cells also secrete granulocyte‐macrophage colony‐stimulating factor (GM‐CSF), which significantly promotes the proliferation and maturation of microglia.^[^ ^181^ ^]^ GM‐CSF binds to GM‐CSFR receptors on microglia, activating the JAK/STAT signaling pathway, which enhances microglial proliferation and survival.^[^ ^182^ ^]^ Smith et al. found that GM‐CSF increases microglial activity, enabling them to efficiently clear apoptotic cells and debris during development.^[^ ^183^ ^]^ Additionally, GM‐CSF facilitates microglial expansion during inflammatory responses, allowing them to rapidly meet the immune demands of the nervous system.^[^ ^184^ ^]^ Thus, GM‐CSF signaling is critical for regulating microglial quantity and activity and maintaining immune homeostasis in the CNS.

IL‐1β, secreted by the vascular system, also plays a vital role in microglial activation and functional regulation.^[^ ^185^ ^]^ IL‐1β binds to IL‐1 receptors on microglia, activating the NF‐κB signaling pathway and triggering immune responses.^[^ ^186^ ^]^ During development, moderate levels of IL‐1β promote microglial activation, enabling them to clear unnecessary synapses and cellular debris, thereby contributing to neural network remodeling.^[^ ^57c^ ^]^ However, excessive IL‐1β signaling can lead to the overactivation of microglia, resulting in inflammatory responses that harm neuronal health.^[^ ^187^ ^]^ Therefore, IL‐1β has a dual role in microglial activation, and the vascular system finely regulates its levels to ensure appropriate immune functions of microglia in the CNS.

Additionally, the vascular system regulates the anti‐inflammatory properties of microglia through the secretion of TGF‐β.^[^ ^188^ ^]^ TGF‐β binds to TGF‐β receptors on microglia, activating the SMAD signaling pathway to suppress excessive microglial activation and shift them into an anti‐inflammatory state.^[^ ^189^ ^]^ During normal development and neuroprotection, TGF‐β signaling reduces microglial inflammatory responses and promotes stability in the neural environment.^[^ ^190^ ^]^ By controlling TGF‐β release, the vascular system effectively modulates microglial activity, allowing them to perform clearance functions without causing excessive neuronal damage.

**Figure 4:**
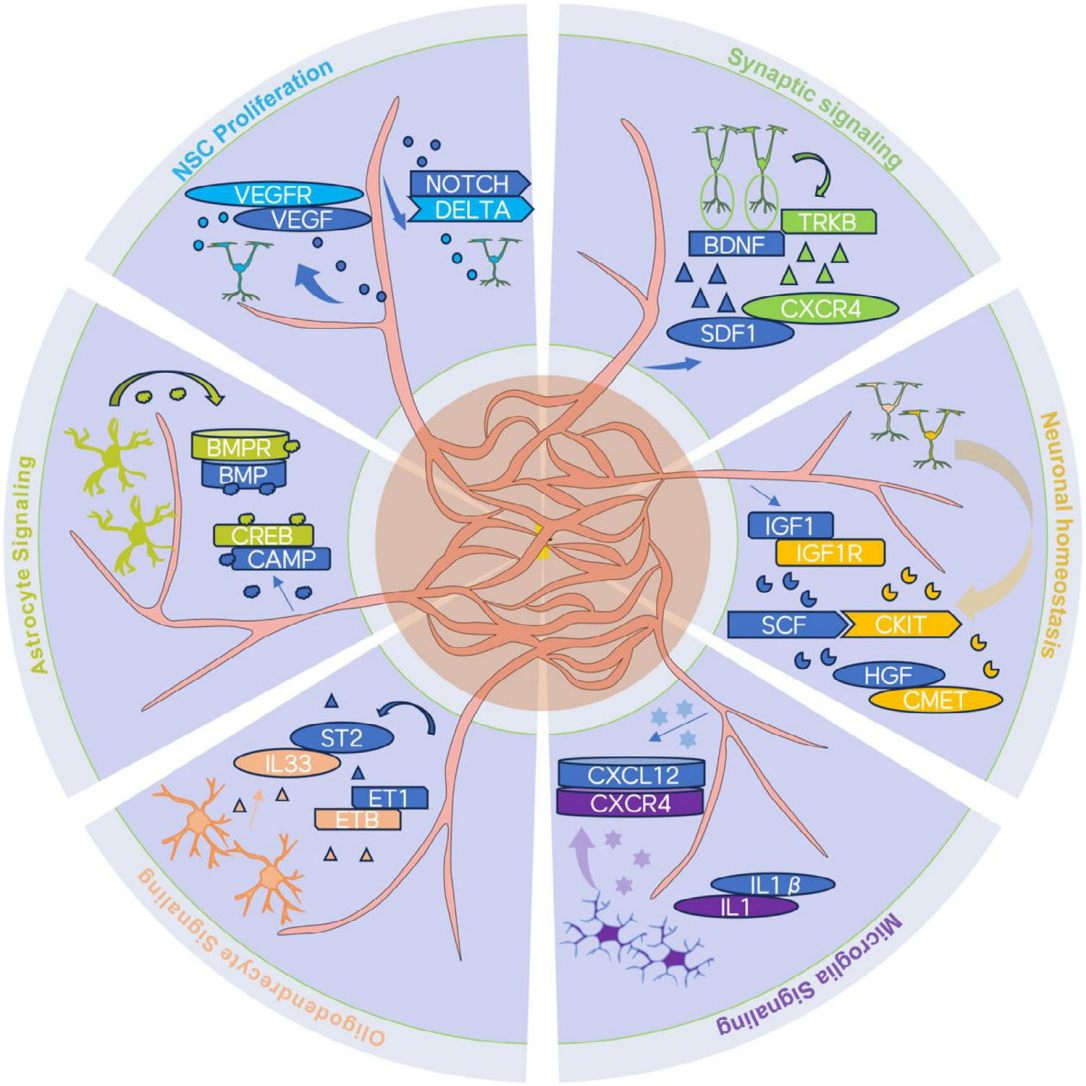
Role of blood vessels in neural development. Blood vessels interact with NSCs via VEGF‐VEGFR, and Notch‐Delta signaling to regulate proliferation and maintenance. Synaptic signaling and neuronal connectivity are influenced by blood vessel‐derived signals such as BDNF‐TrkB and CXCR4‐SDF1. Neuronal homeostasis is supported by IGF1‐IGF1R, SCF‐cKIT, and HGF‐cMET pathways. Astrocyte differentiation and myelination involve BMP‐BMPR and cAMP‐CREB signaling. Oligodendrocytes respond to blood vessel cues through ST2‐IL33 and ET1‐ETB signaling, which also mediate inflammatory responses. Microglia activation and immune surveillance are regulated by IL1β‐IL1 and CXCL12‐CXCR4 pathways. The central neural network emphasizes the integrative role of blood vessels in coordinating cellular functions in the nervous system.

In summary, the vascular system regulates microglial migration, proliferation, activation, and anti‐inflammatory properties through specific signaling molecules such as CXCL12, GM‐CSF, IL‐1β, and TGF‐β. These molecular mechanisms ensure that microglia perform appropriate immune functions during development and homeostasis, providing the necessary immune support and stability for the nervous system. These insights reveal the intricate interactions between the vascular system and microglia, offering critical scientific foundations for understanding CNS health and immune balance, as shown in **Figure** **4**.

## 5 Conclusions 

The formation of a fully functional brain requires not only the regulation of various key genes but also precise cooperation between various cells within the brain. Brain development is a precisely regulated process, and the development of various cells exhibits spatiotemporal specificity, but the developmental process also has overlapping time points. The core processes of brain development include the proliferation and differentiation of NSCs, the formation of blood vessels and BBBs, and the maturation of microglia. In these processes, the loss of key genes in specific tissue types of cells not only leads to their own developmental failure but also causes changes in the surrounding microenvironment, which further affects the development of other tissues. These changes in the microenvironment include the secretion of cytokines and cellular metabolites such as lactate, which can alter the microenvironment in which cells are located. The failure of various types of cell development often leads to abnormal behavior after birth, which is essential for understanding the etiology of neurodevelopmental defects.

## 6 Perspectives

Significant progress has been made in the field of neurodevelopment research in recent years, yet several critical challenges remain to be addressed.

The first key area is the integration of intrinsic genetic programs and extrinsic environmental cues. While considerable advances have been achieved in understanding genetic and epigenetic regulation, the interplay between intrinsic genetic programming and external environmental signals remains poorly defined. Future studies should leverage multi‐omics approaches, such as single‐cell sequencing and spatial transcriptomics, to construct spatiotemporal, multidimensional regulatory networks underlying neurodevelopment. These efforts could elucidate how specific environmental factors, such as maternal immune activation and nutritional status, modulate neuronal fate and brain function via intrinsic genetic programs.

The second critical aspect involves the dynamic transitions and regional heterogeneity of microglial functions. As pivotal regulators of neurodevelopment, the mechanisms underlying microglial heterogeneity and dynamic behaviors remain incompletely understood. Employing real‐time imaging technologies and advanced data analytics will be essential for dissecting microglial dynamics in specific neurological disease models, including autism spectrum disorder and schizophrenia. This approach could clarify their contributions to disease pathogenesis during development.

A third area requiring further investigation is the multiscale interaction between vasculature and neurodevelopment. Cerebral vasculature not only provides metabolic support to the developing nervous system but also directly influences the development of neurons and glial cells through the secretion of specific signaling molecules. However, the spatial and cell‐type‐specific regulatory mechanisms of vascular signals are still poorly characterized. Future research should utilize high‐resolution imaging modalities, such as the integration of optical microscopy with electron microscopy, to probe the structural and functional relationships within the neurovascular unit.

A third area requiring further investigation is the multiscale interaction between vasculature and neurodevelopment. Brain vasculature not only provides essential metabolic support for the developing nervous system but also actively regulates the proliferation, migration, and differentiation of neurons and glial cells through the secretion of specific signaling factors. However, the mechanisms by which vascular signals influence neural development in a spatially regulated and cell‐specific manner remain incompletely understood. Advances in high‐resolution imaging techniques, particularly the integration of optical microscopy with electron microscopy, offer powerful tools for elucidating the structural and functional coupling within the neurovascular unit. Moreover, combining high‐resolution imaging with emerging volumetric techniques, such as volume electron microscopy and optical tissue clearing, holds the potential to map the three‐dimensional neurovascular networks of the developing brain.

Another promising direction is the application of machine learning and artificial intelligence (AI) in neurodevelopmental studies. The rapid growth of multi‐omics datasets and imaging technologies has led to an exponential increase in the volume of data. Advanced AI and machine learning techniques, coupled with biophysical modeling, could offer new avenues to decode the complex interplay of gene expression, cellular behaviors, and network connectivity during neurodevelopment. For instance, training deep learning models to predict developmental trajectories under various genetic and environmental interventions could provide robust tools for advancing precision medicine.

Finally, there is an urgent need to strengthen the integration of basic neurodevelopmental research with clinical applications and disease models. Translational efforts should focus on leveraging advanced platforms, such as brain organoids and humanized mouse models, to investigate the mechanisms underlying gene‐environment interactions in NDDs. These approaches could facilitate the development of novel multi‐pathway therapeutic strategies, accelerating the early diagnosis and personalized treatment of conditions such as developmental epilepsy and psychiatric disorders. In addition, genetic knockout models have greatly advanced our understanding of neurodevelopment, but phenotypic outcomes are influenced by genetic compensation, redundancy, and cell‐type vulnerability. Germline knockouts often trigger compensatory mechanisms that mitigate gene loss, while conditional knockouts, by targeting specific cells or developmental stages, may bypass compensation and produce more pronounced or context‐dependent phenotypes. This distinction is crucial when extrapolating findings to human NDDs, which typically involve complex genetic interactions rather than single‐gene deletions. Conditional knockouts offer precise spatial and temporal gene deletion but may not fully capture the broader effects seen in germline models. A gene regulating neuronal differentiation across multiple brain regions may exhibit severe defects in a germline model but only localized or absent phenotypes in a conditional model. Furthermore, selective gene deletion can lead to artificial phenotypes due to cellular stress, aberrant compensation, or non‐physiological outcomes. Some signaling pathways may become dysregulated in conditional models simply due to the lack of systemic developmental adaptation. Human NDDs often involve polygenic and environmental factors, making them distinct from single‐gene knockout models in animals. While conditional knockouts provide valuable insights into cell‐type‐specific gene functions, they may not fully capture the complexity of human neurodevelopment. Future studies should integrate findings from both conditional and germline models, alongside human genetic research, to improve relevance. Comparative studies, single‐cell and multi‐omics approaches, and patient‐derived iPSC or organoid models will help refine interpretations and provide a more comprehensive understanding of neurodevelopmental mechanisms.

In conclusion, the future of neurodevelopment research will require interdisciplinary collaboration across molecular biology, imaging technologies, AI, and clinical translation. By systematically unraveling the synergistic roles of intrinsic genetic programs and extrinsic environmental cues, these efforts will not only advance our understanding of fundamental brain development but also pave the way for early intervention and therapeutic strategies for NDDs.

## Conflict of Interest

The authors declare no conflict of interest.

## Author Contributions

Y.W., S.Z., and H.J. contributed equally to this work. Y.W. and J.J. conceived the manuscript. Y.W., S.Z., and H.J. drafted the manuscript, drew the figures, and summarized the tables. Y.W. and J.F. discussed the concepts of the manuscript. J.J. supervised the project and secured funding.
